# Trends of influenza vaccination coverage in pregnant women: a ten-year analysis from a French healthcare database

**DOI:** 10.1038/s41598-022-11308-3

**Published:** 2022-05-03

**Authors:** Mélodie Corbeau, Aurélien Mulliez, Chouki Chenaf, Bénédicte Eschalier, Olivier Lesens, Philippe Vorilhon

**Affiliations:** 1 Department of General Practice, Faculty of Medicine, University Clermont Auvergne, 28 Place Henri Dunant, 63001 Clermont-Ferrand, France; 2 Biostatistics Unit (Clinical Research and Innovation Department), University Hospital Clermont-Ferrand, Clermont-Ferrand, France; 3 Service de Pharmacologie Médicale, Centres Addictovigilance et Pharmacovigilance, Université Clermont Auvergne, CHU Clermont-Ferrand, Clermont-Ferrand, France; 4 Université Clermont Auvergne, INSERM, U1107 “Neuro-Dol”, Clermont-Ferrand, France; 5 Infectious and Tropical Diseases Department, CHU Clermont-Ferrand, Clermont-Ferrand, France; 6 Université Clermont Auvergne, ACCePPT, Clermont-Ferrand, France

**Keywords:** Disease prevention, Health policy

## Abstract

Pregnant women have a high risk of severe influenza, associated with obstetrical complications. The World Health Organization (WHO) has recommended influenza vaccination for all pregnant women since 2012. The vaccination coverage remains low worldwide, and in Europe, due to a lack of proposition from the health care providers, and a high refusal rate from the women. The primary aim of this study was to estimate the influenza vaccination coverage (IVC) in a population of pregnant women in France, and to analyse its evolution from 2009 to 2018. The secondary objective was to describe the vaccinated population and to find determinants associated with the vaccination. This retrospective cohort study is based on the EGB French health care database, a representative sample of the French population containing data from the health insurance system. All pregnant women who delivered medically or spontaneously over the 2009–2018 period were included. In the 2009–2018 period, only 1.2% pregnant women were vaccinated against influenza (n = 875/72,207; 95% CI 1.14–1.30). The IVC slightly increased after the 2012 WHO recommendation, from 0.33 to 1.79% (p < 0.001) but remained extremely low (4.1% in 2018). Women younger than 25 years old had a low coverage (0.6%) whereas women over 35 years old were more likely to get the influenza vaccine (1.7%; OR: 2.82, 95% CI 2.14–3.71). The vaccination behavior was not influenced by multifetal pregnancy or parity, but socio-economically deprived women were less likely to be vaccinated (OR: 0.81, 95% CI: 0.67–0.98). Women with pre-existing medical conditions had an overall higher vaccination rate (2.5%; OR: 2.32, 95% CI: 1.94–2.77). The vaccine was mainly prescribed by family physicians (58%). Influenza vaccination in pregnant women in France remains very low, particularly in younger, healthy women, and measures such as information campaigns towards pregnant women and studies of the knowledge, attitudes, and practices of the health care professionals need to be undertaken to improve the coverage.

## Introduction

Influenza is an acute respiratory infection caused by *Myxovirus influenza*, a virus whose subtypes A and B are responsible for seasonal outbreaks worldwide. The World Health Organization (WHO) estimates at a billon the number of influenza cases every year, 3 to 5 million severe cases and 290,000 to 650,000 deaths^1^.

Pregnant women are more likely to develop a severe form of influenza^2–6^ associated with obstetrical complications such as prematurity and miscarriage^7,8^.

Every year, a vaccine is developed with the latest epidemiological information on the circulating subtype in the southern hemisphere. The efficacy and innocuity of the vaccine during pregnancy have been well established in numerous studies^9–20^, as well as its protective effect for the infant through passive immunization^21–23^. Therefore, it should be prescribed to all pregnant women, to avoid severe forms of influenza and to protect their infant.

In Europe the majority of countries have recommended vaccination for pregnant women, whatever the term of pregnancy^24^, since the WHO issued its recommendation in 2012^25^. In France, the influenza vaccination has been recommended for pregnant women since 2009 (in the 2nd and 3rd trimester) and generalized in 2012 to all stages of pregnancy^25,26^. Prioritized during the influenza A(H1N1) outbreak, pregnant women were reluctant to get vaccinated, with an estimated pandemic influenza vaccination coverage (IVC) of 12.8%^27^.

A report issued by the European Centre for Disease Prevention and Control (ECDC) in 2017 showed that the IVC in pregnant women in Europe remained low and uneven, from 0.3 to 56.1% in 2014–2015 and was not monitored by most of the member states, including France^24^. A few studies all based on classical surveys with different methodologies estimated the IVC in France to be between 5.4 and 26%^27–30^. In order to avoid the usual biases arising from classical surveys (selection bias and generalization of the findings), leading to a great variability in coverage estimates, and since the influenza vaccination is prescribed and fully reimbursed by the social security, we propose to use the French national healthcare insurance database to provide estimates of the IVC in pregnant women.

In this study we sought to analyze the evolution of the annual IVC in pregnant women in France from 2009 to 2018, to evaluate the impact of the WHO 2012 recommendation^25^ on vaccination behavior, ad to identify determinants of influenza vaccination uptake in pregnancy.

## Methods

### Data source and design

A repeated annual cross-sectional study was performed by using data from the “Echantillon Généraliste des Bénéficiaires” (EGB), a representative sample of the French population, from January 1, 2009 to December 31, 2018. The EGB database contains the healthcare data of about 660,000 people, representing 1/97th of the French population. This sample of insured patients is randomized, anonymized and representative of the health care protected French population^31,32^. EGB contains sociodemographic information on the beneficiaries, as well as data on various health expenses reimbursed by the French healthcare insurance. That includes prescribed medications (identified according to their anatomical therapeutic chemical class, ATC), consultation with medical and para-medical professional, in private practice as well as public hospital, hospitalization and laboratory expenses. We can also find data on chronic diseases such as ALD (long-term disease) codes, diagnosis and medical history coded during hospitalization using ICD-10 (international classification of diseases, tenth revision) and medical procedures referenced according to the CCAM (common classification of medical acts).

The EGB database is a validated tool for pharmacoepidemiological studies in France and has already proven to be a reliable tool to assess vaccination coverage for other reimbursed vaccines^33–36^.

### Study population

All women who had data recorded on a delivery, vaginal birth, or caesarian section in the EGB database, during the period from January 1, 2009 to December 31, 2018 were included. The exclusion criteria were unwanted pregnancy and denial of pregnancy. We did not exclude pregnancies that resulted in stillbirth, or therapeutic abortion after 22 weeks of gestation.

The corresponding ICD 10 diagnosis codes and CCAM medical procedures are listed in Supplementary Tables 1–3.

### Exposure

Influenza vaccine exposure of the pregnant women included was identified through the reimbursement for a seasonal influenza vaccine in the EGB database, up to nine months before the date of their delivery, using two codes indicating dispensation (ATC J07BB and PRS_NAT 3331). We assumed that a vaccine purchased was injected afterwards and we calculated the IVC based on the number of pregnant women who had a reimbursed influenza vaccine.

To evaluate the impact of the WHO 2012 recommendation^25^ on vaccination in the first trimester, we formed 3 subgroups of women according to their term of pregnancy during the vaccination campaign that takes place from October to January. Women who gave birth from November to February were labeled T3 as they were in their third trimester during the vaccination campaign. Likewise, those who gave birth from March to May were labelled T2, and those who gave birth from June to August were labeled T1.

### Collected data

The collected data concerned were pre-existing medical condition, age, parity, CMUc (universal complementary healthcare insurance), and prescriber’s specialty.

We used the French influenza vaccination recommendation to select women with pre-existing conditions who would have been targeted by the vaccination campaign outside of their pregnancy. Various conditions were included, and grouped in pulmonary, cardiac, neurological, renal, and hepatic disease, diabetes, and immunodeficiency (including HIV, stem cell and all organ transplantations, inflammatory and autoimmune diseases, hematological disease such as sickle cell disease and cancer).

Data were extracted from the EGB database using the CCAM, ICD-10, or long-term chronic disease codes (Supplementary Tables 5–7).

CMUc was used as a proxy for precarity since it is an insurance available only to patients with low income, to insure their access to healthcare free of charge. The women affiliated with the CMUc were considered socioeconomically deprived.

### Data analysis

The population was described by means and standard deviations for continuous data and by numbers and percentages for categorical data.

Analysis of influenza vaccination of pregnant women was carried out using Chi squared test (or Fisher’s exact test when appropriate) for categorical data and using Student’s t-test for continuous data. Odds ratio (of vaccination) between groups are presented with their 95% confidence interval.

To validate the reliability of our data and the computation method of IVC, we compared the IVC calculated through EGB in patients over 65 years old to French official influenza vaccination data (*Santé Publique France*, SPF).

All tests were two-sided. A p-value < 5% was considered statistically significant, but the interpretations were based-on size of differences with clinical view (rather than just regarding the p-value), as significant tests do not always reflect clinically relevant differences when involving large samples. All analyses were performed using SAS Enterprise Guide (SAS Institute Inc, Cary, NC, USA).

### Ethics approval

There was no requirement for ethical approval for this study. The EGB database guarantees the confidentiality and anonymity of all data (agreement by the French Data Protection Authority, CNIL, June 14, 2005).

The use of the EGB database for medical research and for this study in particular has been approved and authorized by the French data protection authority (Commission Nationale de l’Informatique et des Libertés, CNIL). This use is conditioned by a specific training with certification that the researchers must follow. Chouki Chenaf has obtained this certification and was allowed to access and analyze the EGB database for this study. Furthermore, there was no requirement for ethical approval for this study. The EGB database guarantees the confidentiality of all data and anonymity (agreement of French data protection authority on June 14, 2005).

## Results

### Validation data

**Figure 1:**
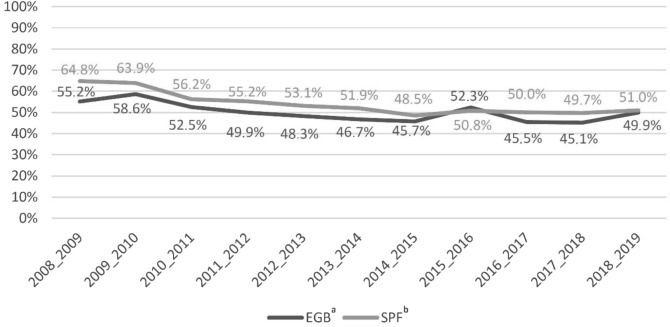
Influenza vaccination coverage in the French population over 65 years old in EGB database and French Public Health data (SPF).

Compared to the nation-wide official influenza vaccination data (SPF) (Fig. 1), data from the EGB database showed a similar trend of influenza vaccination coverage in the French population over 65 years old.

### Influenza vaccination coverage in pregnant women

**Figure 2:**
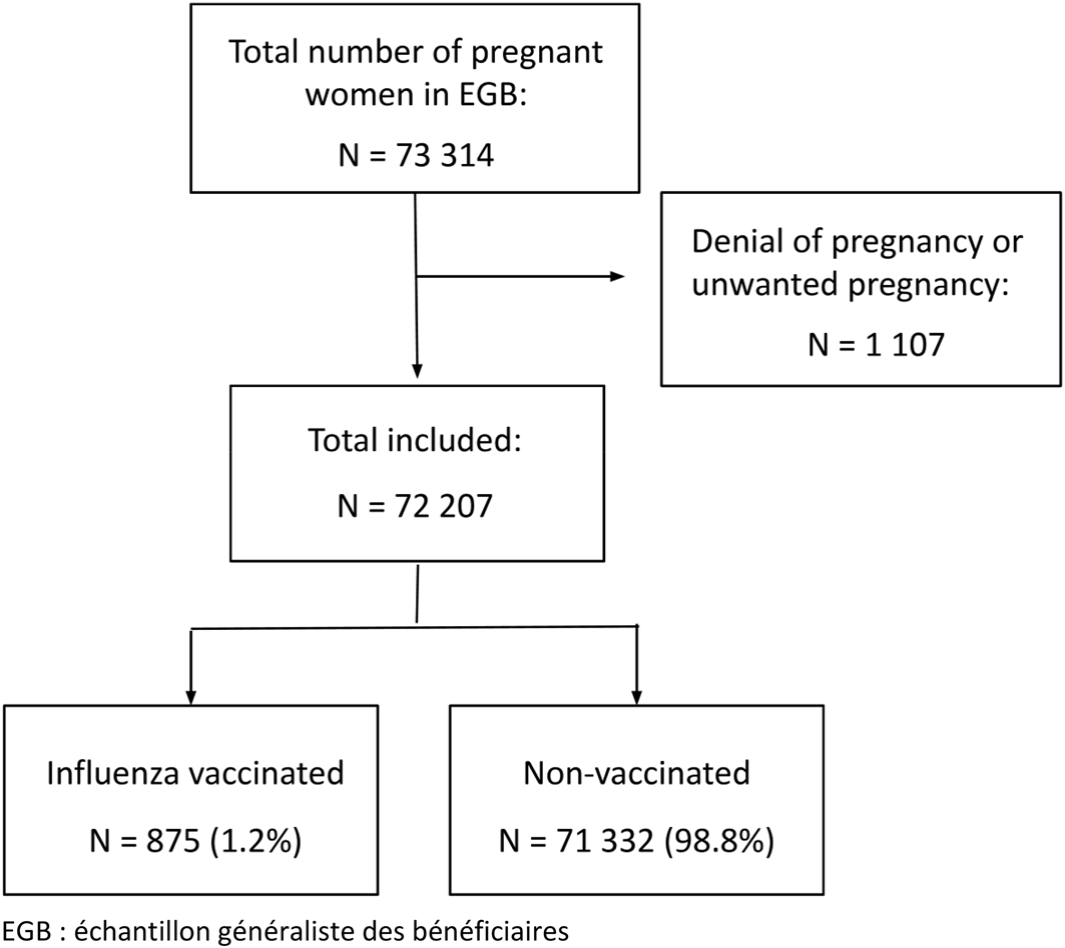
Flow chart of the study.

From January 1, 2009 to December 31, 2018, 73,314 pregnant women were identified in the EGB database. Fifty-two denial of pregnancy and 1055 unwanted pregnancies were excluded from our cohort (Fig. 2). A total of 72,207 pregnant women were included in the study, whose characteristics appear in the Table 1.

**Table 1:** Influenza vaccination and associated factors among pregnant women from 2009 to 2018.

	Unvaccinated	Vaccinated			
	n (%)	n (%)	P value	OR^a^	95% CI^b^
Total	71,332 (100)	875 (100)			
**Age**	30.3 ± 5.40	32.0 ± 5.15	< 0.001		
< 25	10,162 (14.3)	63 (7.2)			
25–29	21,691 (30.4)	215 (24.6)		1.60	1.21–2.12
30–34	23,402 (32.8)	316 (36.1)		2.18	1.66–2.86
> 35	16,075 (22.5)	281 (32.1)		2.82	2.14–3.71
Socioeconomic deprivation (CMUc^c^)	12,512 (17.5)	129 (14.7)	0.030	0.81	0.67–0.98
**Preexisting condition**	5847 (8.2)	150 (17.1)	< 0.001	2.32	1.94–2.77
Pulmonary disease	1198 (1.7)	46 (5.3)	< 0.001	3.25	2.4–4.4
Cardiac disease	1633 (2.3)	45 (5.1)	< 0.001	2.31	1.7–3.13
Neurological disease	163 (0.2)	3 (0.3)	0.487	1.5	0.48–4.71
Renal disease	137 (0.2)	12 (1.4)	< 0.001	7.23	3.99–13.09
Sickle-cell disease	128 (0.2)	6 (0.7)	< 0.001	3.84	1.69–8.73
Diabetes	392 (0.5)	36 (4.1)	< 0.001	7.77	5.49–11
Immunodeficiency	1057 (1.5)	35(4)	< 0.001	2.77	1.96–3.91
Hepatic disease	293 (0.4)	9 (1.0)	0.005	2.52	1.29–4.91
Obesity	2561 (3.6)	39 (4.5)	0.171	1.25	0.9–1.73
Multifetal pregnancy	1394 (2.0)	21 (2.4)	0.344	1.23	0.8–1.9
**Parity**			0.820		
1	34,698 (48.6)	429 (49.0)		1.02	0.89–1.17
> 2	36,634 (51.4)	446 (51.0)		0.98	0.86–1.12
Prematurity	6307 (8.8)	70 (8.0)	0.383	0.9	0.7–1.15
Still birth	203 (0.3)	1 (0.1)	0.345	0.4	0.06–2.86
C-section	14,534 (20.4)	197 (22.5)	0.119	1.32	1.13–1.55
Therapeutic abortions	214 (0.3)	1 (0.1)	0.316	0.38	0.05–2.71

^a^OR: odds ratio calculated with Wald test.

^b^CI: confidence interval.

^c^CMUc: couverture mutuelle universelle complémentaire ( universal complementary healthcare insurance).

**Figure 3:**
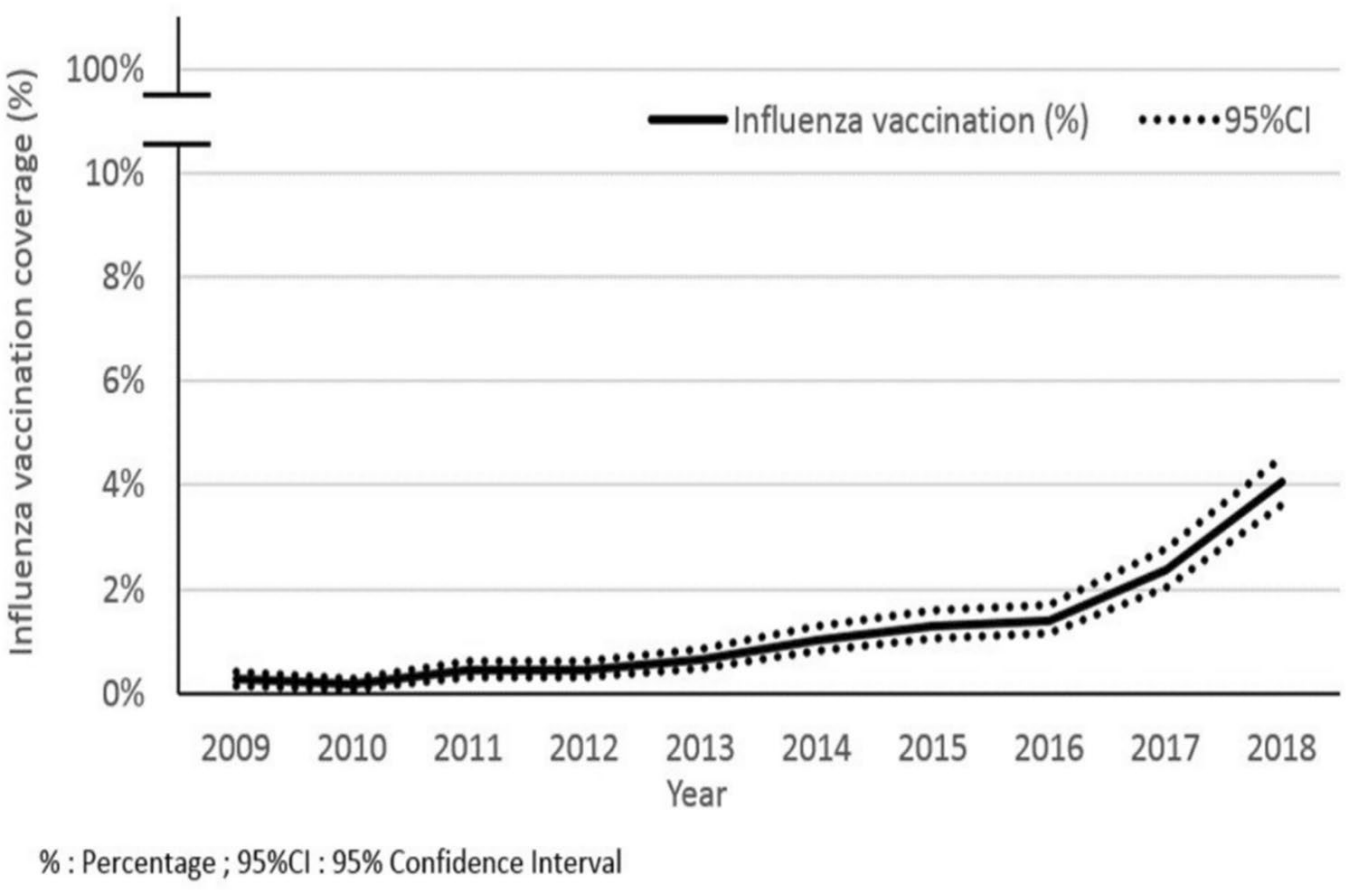
Evolution of the influenza vaccination coverage from 2009 to 2018.

The overall influenza vaccination coverage (IVC) was only of 1.21% in the 2009–2018 period (n = 875/72,207; 95% CI 1.14–1.30). The IVC slightly increased after the 2012 recommendation, from 0.33% in the 2009–2012 period, to 1.79% in the 2013–2018 period (p < 0.001) (Fig. 3).

Vaccinated women were older, less socially deprived, and had more pre-existing medical condition. There was no difference regarding the pregnancy outcome in the vaccinated and non-vaccinated population.

The mean gestational age at delivery was 38.9 weeks in both vaccinated and non-vaccinated populations. The mean gestational age of vaccination was 22.9 weeks ± 9.7.

**Figure 4:**
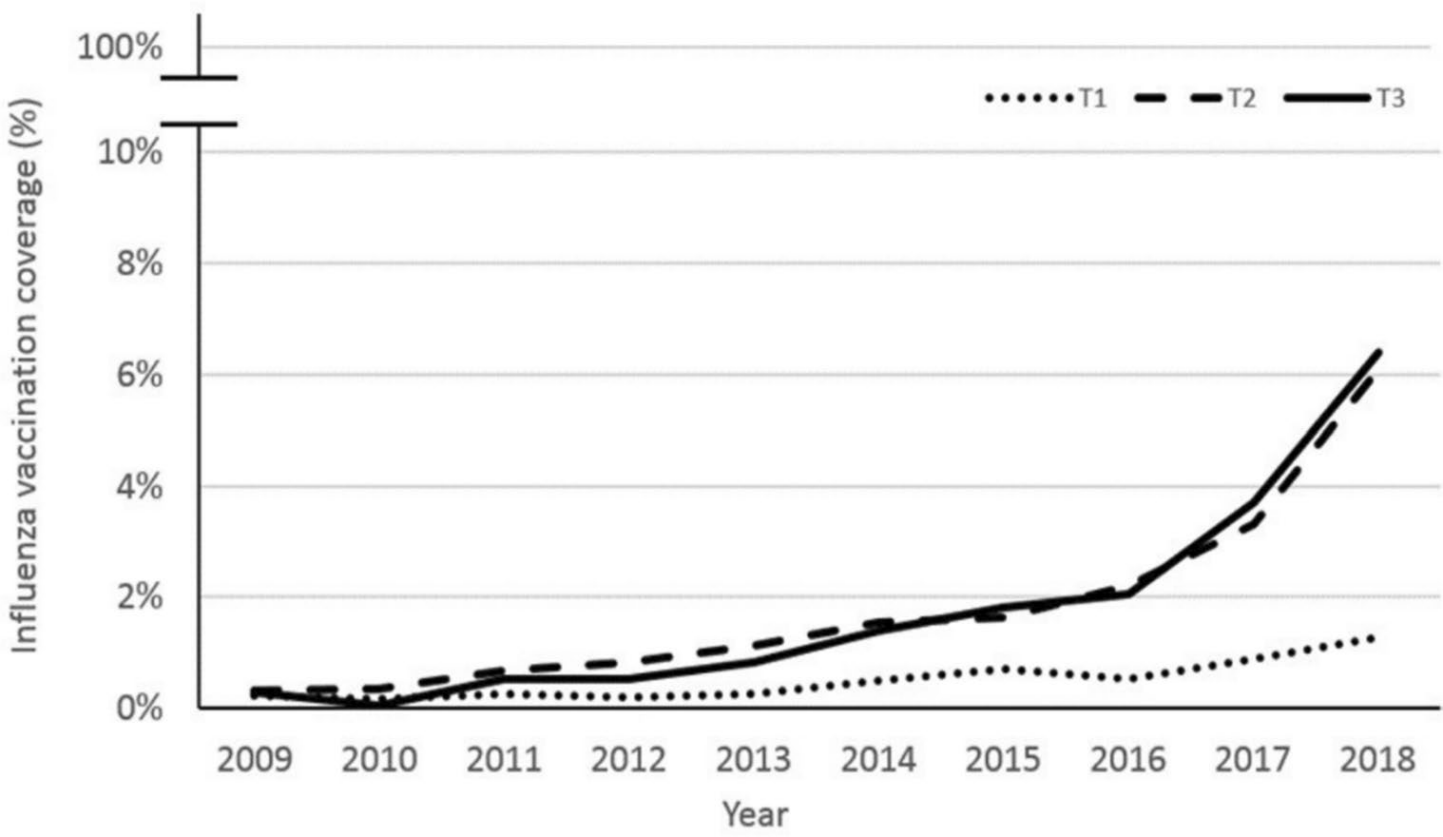
Evolution of influenza vaccination coverage by trimester of pregnancy during the vaccination campaign.

The identified prescribers were mainly family physicians (58%), followed by gynecologists (21%) and midwives (4%). Some specialists sporadically prescribed the vaccine (total of 5%) and 12% were dispensed directly by the pharmacists. Women were most likely to be vaccinated if they were in their 2nd or 3rd trimester of pregnancy during the official vaccination campaign (Fig. 4), even after the 2012 recommendation was issued.

### Influenza vaccination determinants

The vaccinated population was slightly older than the non-vaccinated one (respectively 32.0 and 30.3 years old, p < 0.001) and the vaccination rate increased proportionally with the age of the population (see Table 1). Women younger than 25 years old had a low coverage (0.6%) whereas women over 35 years old were more likely to get the influenza vaccine (1.7%; OR: 2.82, 95% CI 2.14–3.71). Socio-economically deprived women were less likely to be vaccinated (OR: 0.81, 95% CI: 0.67–0.98). Women with pre-existing medical conditions at risk for severe influenza had an overall higher vaccination rate (2.5%; OR: 2.32, 95% CI: 1.94–2.77). Of all the disease, renal disease and diabetes were associated with the highest vaccination rates (respectively 8.1% and 8.4%), and obesity with the lowest (1.5%).

The vaccination behavior was not influenced by multifetal pregnancy (OR: 1.23, 95% CI 0.8–1.9) or by parity: primiparous and multiparous women had the same vaccination rate (1.2%).

## Discussion

### Main results

Our primary endpoint was to analyze the evolution of the IVC in pregnant women in France from 2009 to 2018 using the French national healthcare database. We showed that the IVC increased slightly since the 2012 recommendation but remained extremely low (only up to 4.1% in 2018), thus keeping pregnant women and their fetuses at risk of complications from influenza infections. In addition, the coverage for women who started their pregnancy during the vaccination campaign remained lower than those of women in their 2nd and 3rd trimester, thus questioning the impact of the recommendation, that encouraged the vaccination of women at all term of pregnancy. The IVC was higher for older women, and women with pre-existing condition at risk for severe influenza, and lower in socially deprived women as often described in other studies^30,37–39^.

The vaccines were mainly prescribed by family physicians, even though gynecologists are usually the main healthcare providers during the pregnancy (68% according to Descamps et al.)^30^. It is most likely due to the fact that family physicians are traditionally in charge of immunizations.

### Comparison with data from the literature

In Europe, the European Centre for Disease prevention and Control (ECDC) regularly compares the coverage rate in member states. In 2016–2017 the IVC was known for 9 member states, and ranged from 0.5% in Slovenia to 58.6% in the UK (median 25.0%)^40^. In other northern countries similar rates were found, 48.1% in the USA^41^, and 30.8% in New Zealand in 2018^39^.

Most data available in France are based on surveys and range from 7 to 26%.

In 2014, Gaudelus et al. found an IVC in pregnant women of 7% through an online survey based on self-administered questionnaires^28^. In 2015, Loubet et al. found a IVC of 26% through an online survey, with a small non-representative sample and many selection bias^29^. Then Descamps et al. calculated an IVC of 7.4% based on the National Perinatal Survey, a large national study conducted in March 2016^30^. This data was obtained through a declarative method where women were interviewed by a healthcare professional in the postpartum ward before discharge. Our study finds a IVC of 2.4% for women who gave birth in the same period of time (vs 7.4%) and part of this difference can be explained by a social desirability bias^42^ and recall bias that may over-estimate the IVC in any declarative vaccination evaluation. Hu et al. compared survey data and administrative data in children’s vaccination, to evaluate the impact of recall bias by their mother. The results showed the frequency of over-reporting of vaccination in the survey data, up to 6.6% for DPT vaccine, linked to social desirability bias^43^.

### Influenza vaccination determinants

The determinants of low IVC in pregnant women have been well studied and two main obstacles to the vaccination are identified. The first obstacle is the lack of proposition from healthcare providers worldwide. In France, as in many other countries, only 25% of women are offered the vaccination^28,44–47^, when Canada or the United States offers it to 75% of women^48,49^. It is often linked to the vaccination status of the healthcare provider, that is also known to be very low^50,51^. The second obstacle is the refusal of the vaccine by pregnant women who either fear an impact of the vaccination on the pregnancy or underestimate the gravity of severe influenza. The refusal rate in France is 68%^28,30^.

The education of healthcare professional is key to improve IVC in pregnant women^45^. Psarris et al. led a study in Greece in 2018 where the proposition rate rose from 27 to 100% and the IVC from 14 to 94% after a simple information campaign towards healthcare professionals^44^.

Another determinant is the availability of the vaccine. Alessandrini et al. led a cross-sectional multicenter study in three maternities in Paris, where vaccination was available immediately during prenatal consultations, free of charge. The coverage increased from 0 to 35.4%^52^.

The 2009 A (H_1_N_1_) pandemic also had a negative impact on influenza vaccination. A study led in 2010 and 2011 showed that the IVC decreased significantly the years after the A (H_1_N_1_) pandemic in France^53^. The overall pandemic vaccination coverage was low in general population (11.1%), about half the seasonal IVC (20%), and similar in patients at risk of severe influenza (12.2%)^27^. French authorities recommended pandemic A (H_1_N_1_) vaccination with a single dose of an adjuvanted-free vaccine (Panenza®) for all pregnant women after the first trimester. and the pandemic vaccination coverage was similar to the at-risk population (12.9%).

The burden of A (H_1_N_1_) influenza was not as heavy as anticipated in France, with about 300 death and 1300 patients with serious forms of influenza^54^, much lower than expected. The disproportion between the impact of the epidemic and the mass vaccination campaign that was conducted, added to the many controversies and debate around the effectiveness of the vaccine led to a prolonged confidence crisis in seasonal influenza vaccination, and in a global mistrust of the French health authorities^36^.

Although pregnant women are at increased risk for severe COVID-19, the same doubts and mistrust are found today in France regarding vaccination^55^.

### Strengths and limitations of the study

The main strengths of the study are the size of the study population and the reproducibility of the calculation of the IVC through the EGB database. Our administrative method is reliable to assess a vaccination coverage in a limited time, on a large representative sample, with less bias than a declarative study. Our IVC results on patients over 65 years old are similar to the French official data, validating the use of the database to evaluate the IVC.

Its limitations are those of the database, that can only record reimbursed vaccines, leaving out over the counter purchase and workplace vaccination. Influenza vaccination is recommended for patients over 65 years old, with chronic disease, obese patients, and pregnant women and their vaccine is supposed to be prescribed and fully reimbursed. In addition, anyone can buy it over the counter, for a reasonable price. We assumed that a pregnant woman would turn to her physician before making any health decision during her pregnancy and would have the prescription for the vaccine. However, women who purchased the vaccine over the counter on their own initiative or got the injection in their workplace would have been falsely classified as “unvaccinated” in our study.

Even though our data cannot be fully exhaustive due to this easy access to the vaccine outside of the traditional health care pathway, our results are useful for analysis and comparison purposes.

We identified some limitations in our secondary endpoints:

Pre-existing medical condition were assessed through the existence of a long-term disease data claim, or hospital discharge quotation of an act or a pathology, as well a medication prescribed related to the pathology. We may have overlooked woman that had a condition that put them at risk of a severe influenza, but who requires no specific treatment and had no record of it in their files.

Parity was estimated with the data available in EGB and is indicative. The data collected goes back to 2005, and any pregnancy occurring before that date will not have been taken into account, as well as any prior pregnancy that may have taken place abroad.

We chose to define socioeconomic deprivation by the CMUc affiliation, a specific health insurance with full coverage for low-income patients. However, socioeconomic deprivation is a wider concept that includes criteria such as insecure employment, lack of familial support, and reduced access to culture, sport or vacation^56^. The EPICES score (Evaluation of precariousness and Inequalities in Health Examination Centers) is more reliable to describe socioeconomic deprivation^57,58^. Unfortunately, it requires data that are not available on the EGB database.

### Perspectives for research and healthcare

A study evaluating the knowledge, attitudes, and practices of healthcare professionals regarding influenza vaccination in France could help to improve the IVC, taking into consideration the global reluctance towards vaccination in France. The IVC could be improved by targeted information campaigns towards all healthcare professionals involved with pregnancy care, and towards women in general.

A national information campaign was issued on French television to promote influenza vaccination during the winter of 2020–2021, and pregnant women were listed as a priority. The impact of such campaigns could be monitored, by issuing IVC data yearly.

Our evaluation of IVC in pregnant women with the EGB database may lead the way to other vaccination evaluations, such as pertussis vaccination coverage. A pertussis booster is often needed to properly protect the newborn from a severe infection, and an evaluation of the vaccination coverage could help improve this protection.

Finally, from the beginning of the COVID-19 pandemic, pregnant women were considered at high-risk of severe forms of the disease, and they were included in specific clinical trial to demonstrate the mRNA vaccine safety and efficacy^59^. The vaccination, while limited at first to women with comorbidities, or with high-exposure professions, is now recommended for all pregnant women in their 2nd and 3rd trimester. The large-scale communication towards healthcare professionals on the necessity of COVID-19 vaccination during pregnancy may have a positive impact on the IVC.

## Conclusion

The influenza vaccination coverage in pregnant women has slightly increased in France since the 2012 WHO recommendation, particularly during the 2nd and 3rd trimester of pregnancy, but remains extremely low (4.1% in 2018). Older women and women with a pre-existing condition at risk for severe influenza were more likely to be vaccinated whereas socio-economic deprived women had a lower coverage. Parity and multifetal pregnancy did not influence the vaccination behavior. The influenza vaccine was mainly prescribed by family physicians. The influenza vaccination needs to be generalized during pregnancy, and measures such as information campaigns and studies of the knowledge, attitudes and practices of both pregnant women and healthcare professionals need to be undertaken to improve the coverage.

## Supplementary Information

Supplementary Tables.

## Data Availability

References cited are available and accessible to the public.
