# Supplementary material for: Trends of influenza vaccination coverage in pregnant women: a ten-year analysis from a French healthcare database
Source: Sci Rep. 2022 May 3;12:7153. doi: 10.1038/s41598-022-11308-3 (PMC9062868; doi:10.1038/s41598-022-11308-3)
Supplement: Supplementary file 1 — Supplementary Tables. [file 41598_2022_11308_MOESM1_ESM.docx]

# Supplementary material

**Table S1 –** **International Classification of Diseases (ICD-10) Codes for delivery**

| O60 | Preterm labor and delivery |
| --- | --- |
| O601 | Preterm labor with spontaneous preterm delivery |
| O603 | Preterm delivery without spontaneous labor |
| O67 | Labor and delivery complicated by intrapartum hemorrhage, not elsewhere classified |
| O68 | Labor and delivery complicated by fetal distress |
| O680 | Labor and delivery complicated by fetal heart rate anomaly |
| O681 | Labor and delivery complicated by meconium in amniotic fluid |
| O682 | Labor and delivery complicated by fetal heart rate anomaly and meconium in amniotic fluid |
| O683 | Labor and delivery complicated by biochemical evidence of fetal stress |
| O688 | Labor and delivery complicated by other evidence of fetal stress |
| O689 | Labor and delivery complicated by fetal stress, unspecified |
| O69 | Labor and delivery complicated by umbilical cord complications |
| O690 | Labor and delivery complicated by prolapse of cord |
| O691 | Labor and delivery complicated by cord around neck, with compression |
| O692 | Labor and delivery complicated by other cord entanglement, with compression |
| O693 | Labor and delivery complicated by short cord |
| O694 | Labor and delivery complicated by vasa previa |
| O695 | Labor and delivery complicated by vascular lesion of cord |
| O698 | Labor and delivery complicated by other cord complications |
| O699 | Labor and delivery complicated by cord complication, unspecified |
| O755 | Delayed delivery after artificial rupture of membranes |
| O756 | Delayed delivery after spontaneous or unspecified rupture of membranes |
| O757 | Vaginal delivery following previous cesarean section |
| O80 | Encounter for full-term uncomplicated delivery |
| O800 | Single vertex delivery |
| O801 | Single breech delivery |
| O808 | Other single spontaneous delivery |
| O809 | Single spontaneous delivery, unspecified |
| O81 | Single delivery by forceps and vacuum extractor |
| O810 | Low forceps delivery |
| O811 | Mid cavity forceps delivery |
| O812 | Mid cavity forceps V with rotation |
| O813 | Other and unspecified forceps delivery |
| O814 | Vacuum extractor delivery |
| O815 | Delivery by combination of forceps and vacuum extractor |
| O82 | Single delivery by cesarean section |
| O820 | Delivery by elective cesarean section |
| O821 | Delivery by emergency cesarean section |
| O822 | Delivery by cesarean section with hysterectomy |
| O828 | Other single delivery by cesarean section |
| O829 | Delivery by cesarean delivery, unspecified |
| O83 | Other assisted single delivery |
| O830 | Breech extraction |
| O831 | Other assisted breech extraction |
| O832 | Other manipulation-assisted delivery |
| O833 | Delivery of viable fetus in abdominal pregnancy |
| O834 | Destructive operation for delivery |
| O838 | Other specified assisted single delivery |
| O839 | Assisted single delivery, unspecified |
| O84 | Multiple delivery |
| O840 | Multiple delivery, all spontaneous |
| O841 | Multiple delivery, all by forceps and vacuum extractor |
| O842 | Multiple delivery, all by cesarean section |
| O848 | Other multiple delivery |
| O849 | Multiple delivery, unspecified |
| Z370 | Single live birth |
| Z371 | Single stillbirth |
| Z372 | Twins, both liveborn |
| Z373 | Twins, one liveborn, one stillborn |
| Z374 | Twins, both stillborn |
| Z375 | Other multiple births, all liveborn |
| Z376 | Other multiple births, some liveborn |
| Z377 | Other multiple births, all stillborn |
| Z379 | Outcome of delivery, unspecified |

**Table S2 - Common Classification of Medical acts for delivery**

| JQGA002 | Delivery by elective cesarean section, by laparotomy |
| --- | --- |
| JQGA003 | Delivery by cesarean section during labor by laparotomy |
| JQGA004 | Delivery by emergency cesarean section by laparotomy |
| JQGA005 | Delivery by vaginal cesarean section |
| JQGD001 | Single breech delivery |
| JQGD002 | Multiple delivery, primiparous |
| JQGD003 | Single breech delivery with minimal extraction, primiparous |
| JQGD004 | Single breech delivery, primiparous |
| JQGD005 | Single breech delivery, multiparous |
| JQGD007 | Multiple delivery, multiparous |
| JQGD008 | Single breech delivery with minimal extraction, multiparous |
| JQGD010 | Single vertex delivery, primiparous |
| JQGD011 | Manipulation-assisted breech delivery |
| JQGD012 | Single vertex delivery, multiparous |
| JQGD013 | Single breech delivery with maximal extraction, primiparous |

**Table S3 - International Classification of Diseases codes for non-inclusion criteria**

| Z640 | Unwanted pregnancy |
| --- | --- |
| Z33 | Denial of pregnancy |

##### Table S4 - International Classification of Diseases codes for secondary endpoints

| Z641 | Problems related to multiparity |
| --- | --- |
| Z354 | Supervision of pregnancy with grand multiparity |

##### Table S5 - International Classification of Diseases codes for medical risk factors

1. **Chronic pulmonary diseases**

| J45 | Asthma |
| --- | --- |
| J450 | Predominantly allergic asthma |
| J451 | Nonallergic asthma |
| J458 | Mixed asthma |
| J459 | Other and unspecified asthma |
| Z870 | Personal history of diseases of the respiratory system |
| 0995 | Diseases of the respiratory system complicating pregnancy, childbirth, and the puerperium |
| J961 | Chronic respiratory failure |
| J441 | Chronic obstructive pulmonary disease with acute exacerbation, unspecified |
| J448 | Other specified chronic obstructive pulmonary disease |
| J969 | Respiratory failure, unspecified |
| P279 | Unspecified chronic respiratory disease originating in the perinatal period |
| J40 | Bronchitis, not specified as acute or chronic |
| J410 | Simple chronic bronchitis |
| J411 | Mucopurulent chronic bronchitis |
| J418 | Mixed simple and mucopurulent chronic bronchitis |
| J42 | Unspecified chronic bronchitis |
| J448 | Other specified chronic obstructive pulmonary disease |
| J670 | Hypersensitivity pneumonitis due to organic dust |
| J684 | Chronic respiratory conditions due to chemicals, gases, fumes, and vapors |
| M954 | Acquired deformity of chest and rib |
| Q768 | Other congenital malformations of bony thorax |
| Q769 | Congenital malformation of bony thorax, unspecified |
| Q32 | Congenital malformations of trachea and bronchus |
| Q33 | Congenital malformation of lung |
| Q330 | Congenital cystic lung |
| J47 | Bronchiectasis |
| J980 | Diseases of bronchus, not elsewhere classified |
| P271 | Bronchopulmonary dysplasia originating in the perinatal period |
| Q336 | Hypoplasia and dysplasia of lung |
| E84 | Cystic fibrosis |
| E840 | Cystic fibrosis with pulmonary manifestations |
| E848 | Cystic fibrosis with other manifestations |
| E849 | Cystic fibrosis, unspecified |

1. **Cardiac diseases**

| Q20 | Congenital malformations of cardiac chambers and connections |
| --- | --- |
| Q209 | Congenital malformation of cardiac chambers and connections, unspecified |
| Q26 | Congenital malformations of great veins |
| Q231 | Congenital insufficiency of aortic valve |
| Q248 | Other specified congenital malformations of heart |
| Q249 | Congenital malformation of heart, unspecified |
| I110 | Hypertensive heart disease with (congestive) heart failure |
| I130 | Hypertensive heart and renal disease with (congestive) heart failure |
| I1300 | Hypertensive heart and renal disease with heart failure, LVEF ≥ 50 |
| I1302 | Hypertensive heart and renal disease with both (congestive) heart failure and renal failure |
| I1309 | Hypertensive heart and renal disease, unspecified |
| I1320 | Hypertensive heart and renal disease with heart and renal failure, LVEF ≥ 50 |
| I1321 | Hypertensive heart and renal disease with heart and renal failure, LVEF ≥ 40, and <50 |
| I1322 | Hypertensive heart and renal disease with heart and renal failure, LVEF <40 |
| I1329 | Hypertensive heart and renal disease with heart and renal failure, LVEF unspecified |
| I25 | Chronic ischemic heart disease |
| I251 | Atherosclerotic heart disease |
| I252 | Old myocardial infarction |
| I253 | Aneurysm of heart |
| I254 | Coronary artery aneurysm and dissection |
| I255 | Ischemic cardiomyopathy |
| I256 | Silent myocardial ischemia |
| I258 | Other forms of chronic ischemic heart disease |
| I259 | Chronic ischemic heart disease, unspecified |
| I499 | Cardiac arrhythmia, unspecified |
| I426 | Alcoholic cardiomyopathy |
| I427 | Cardiomyopathy due to drugs and other external agents |
| I50 | Heart failure |
| I500 | Congestive heart failure |
| I5000 | Congestive heart failure, LVEF ≥ 50 |
| I5001 | Congestive heart failure, LVEF ≥ 40, and <50 |
| I5002 | Congestive heart failure, LVEF <40 |
| I5009 | Congestive heart failure, LVEF unspecified |
| I501 | Left ventricular failure |
| I5010 | Left ventricular failure, LVEF ≥ 50 |
| I5011 | Left ventricular failure, LVEF ≥ 40, and <50 |
| I5012 | Left ventricular failure, LVEF <40 |
| I5019 | Left ventricular failure, LVEF unspecified |
| I509 | Heart failure, unspecified |
| I517 | Cardiomegaly |
| I38 | Valvular insufficiency of unspecified valve |
| I059 | Mitral valve disease, unspecified |
| I069 | Rheumatic aortic valve disease, unspecified |
| I079 | Tricuspid valve disease, unspecified |
| I080 | Multiple valve diseases |
| I081 | Disorders of both mitral and tricuspid valves |
| I082 | Disorders of both aortic and tricuspid valves |
| I083 | Combined disorders of mitral, aortic and tricuspid valves |
| I088 | Other multiple valve diseases |
| I089 | Multiple valve disease, unspecified |
| I091 | Rheumatic diseases of endocardium, valve unspecified |
| I099 | Rheumatic heart disease, unspecified |
| I238 | Other current complications following acute myocardial infarction |
| I348 | Other nonrheumatic mitral valve disorders |
| I349 | Nonrheumatic mitral valve disorder, unspecified |
| I350 | Aortic valve stenosis |
| I359 | Aortic valve disorder, unspecified |
| I379 | Pulmonary valve disorder, unspecified |
| I44 | Atrioventricular and left bundle-branch block |
| I45 | Other conduction disorders |
| I459 | Conduction disorder, unspecified |
| I471 | Supraventricular tachycardia |
| I472 | Ventricular tachycardia |
| I479 | Paroxysmal tachycardia, unspecified |
| I49 | Other cardiac arrhythmias |
| I498 | Other specified cardiac arrhythmias |
| I499 | Cardiac arrhythmia, unspecified |
| I978 | Other postprocedural disorders of circulatory system, not elsewhere classified |
| Q229 | Congenital malformation of tricuspid valve, unspecified |
| Q231 | Congenital insufficiency of aortic valve |
| Q233 | Congenital mitral insufficiency |
| E854 | Cardiac amyloidosis |
| O994 | Diseases of the circulatory system complicating pregnancy, childbirth, and the puerperium |
| O998 | Other specified diseases and conditions complicating pregnancy, childbirth, and the puerperium |
| Z867 | Personal history of diseases of the circulatory system |
| Z95 | Presence of cardiac and vascular implants and grafts |

1. **Neurological disease**

| I64 | Stroke, not specified as hemorrhage or infarction |
| --- | --- |
| Z8660 | Personal history of diseases of the nervous system and sense organs |
| G459 | Transient cerebral ischemic attack, unspecified |
| I694 | Sequelae of stroke, not specified as hemorrhage or infarction |
| M63 | Disorders of muscle in diseases classified elsewhere |
| G60 | Hereditary and idiopathic neuropathy |
| G71 | Primary disorders of muscles |
| G710 | Muscular dystrophy |
| G712 | Congenital myopathies |
| G713 | Mitochondrial myopathy, not elsewhere classified |
| G72 | Other myopathies |
| G722 | Myopathy due to other toxic agents |
| G724 | Inflammatory myopathy, not elsewhere classified |
| G728 | Other specified myopathies |
| G729 | Myopathy, unspecified |
| A80 | Acute poliomyelitis |
| A800 | Acute paralytic poliomyelitis, vaccine-associated |
| A801 | Acute paralytic poliomyelitis, wild virus, imported |
| A802 | Acute paralytic poliomyelitis, wild virus, indigenous |
| A803 | Acute paralytic poliomyelitis, other and unspecified |
| A804 | Acute nonparalytic poliomyelitis |
| P358 | Congenital poliomyelitis |
| B91 | Sequelae of poliomyelitis |
| G700 | Myasthenia gravis |
| G702 | Congenital and developmental myasthenia |
| G711 | Myotonic disorders |
| G122 | Motor neuron disease |
| J986 | Paralysis of diaphragm |
| S278 | Diaphragm injury |

1. **Renal diseases**

| N009 | Acute nephritic syndrome, unspecified |
| --- | --- |
| N03 | Chronic nephritic syndrome |
| N031 | Chronic nephritic syndrome, focal and segmental glomerular lesions |
| N032 | Chronic nephritic syndrome, diffuse membranous glomerulonephritis |
| N033 | Chronic nephritic syndrome, diffuse mesangial proliferative glomerulonephritis |
| N034 | Chronic nephritic syndrome, diffuse endocapillary proliferative glomerulonephritis |
| N035 | Chronic nephritic syndrome, diffuse mesangiocapillary glomerulonephritis |
| N036 | Chronic nephritic syndrome, dense deposit disease |
| N037 | Chronic nephritic syndrome, diffuse crescentic glomerulonephritis |
| N038 | Other glomerulonephritis |
| N144 | Toxic nephropathy, not elsewhere classified |
| N17 | Acute renal failure |
| N170 | Acute renal failure with tubular necrosis |
| N171 | Acute renal failure with acute cortical necrosis |
| N172 | Acute renal failure with medullary necrosis |
| N18 | Chronic kidney disease |
| N180 | End-stage renal disease |
| N181 | Chronic kidney disease, stage 1 |
| N182 | Chronic kidney disease, stage 2 |
| N183 | Chronic kidney disease, stage 3 |
| N184 | Chronic kidney disease, stage 4 |
| N185 | Chronic kidney disease, stage 5 |
| N188 | Other chronic kidney disease |
| N189 | Chronic kidney disease, unspecified |
| N19 | Unspecified kidney failure |
| N28 | Other disorders of kidney and ureter, not elsewhere classified |
| N280 | Ischemia and infarction of kidney |
| N288 | Other specified disorders of kidney and ureter |
| N289 | Disorder of kidney and ureter, unspecified |
| N29 | Other disorders of kidney and ureter in diseases classified elsewhere |
| N298 | Other disorders of kidney and ureter in other diseases classified elsewhere |
| N990 | Postprocedural renal failure |
| I120 | Hypertensive renal disease with renal failure |
| I129 | Hypertensive renal disease without renal failure |
| I139 | Hypertensive heart and renal disease, unspecified |
| K767 | Hepatorenal syndrome |
| N040 | Nephrotic syndrome with minor glomerular abnormality |
| N042 | Nephrotic syndrome, diffuse membranous glomerulonephritis |
| N043 | Nephrotic syndrome, diffuse mesangial proliferative glomerulonephritis |
| N044 | Nephrotic syndrome, diffuse endocapillary proliferative glomerulonephritis |
| N045 | Nephrotic syndrome, diffuse mesangiocapillary glomerulonephritis |
| N046 | Nephrotic syndrome, dense deposit disease |
| N047 | Nephrotic syndrome, diffuse crescentic glomerulonephritis |
| N048 | Nephrotic syndrome, other morphological lesions |
| N049 | Nephrotic syndrome, unspecified |

1. **Sickle cell disease**

| D57 | Sickle-cell disorders |
| --- | --- |
| D570 | Sickle-cell anemia with crisis |
| D571 | Sickle-cell anemia without crisis |
| D572 | Double heterozygous sickling disorders |
| D573 | Sickle-cell trait |
| D578 | Other sickle-cell disorders |

1. **Diabetes**

| E10 | Type 1 diabetes mellitus |
| --- | --- |
| E108 | Type 1 diabetes mellitus with unspecified complications |
| E109 | Type 1 diabetes mellitus without complications |
| E11 | Type 2 diabetes mellitus |
| E118 | Type 2 diabetes mellitus with unspecified complications |
| E119 | Type 2 diabetes mellitus without complications |
| E14 | Unspecified diabetes mellitus |
| E148 | Unspecified diabetes mellitus with unspecified complications |
| E149 | Unspecified diabetes mellitus without complications |

1. **Immunodeficiency**

| D802 | Selective deficiency of immunoglobulin A [IgA] |
| --- | --- |
| D803 | Selective deficiency of immunoglobulin G [IgG] subclasses |
| D808 | Other immunodeficiencies with predominantly antibody defects |
| D81 | Combined immunodeficiencies |
| D811 | Severe combined immunodeficiency [SCID] with low T- and B-cell numbers |
| D812 | Severe combined immunodeficiency [SCID] with low or normal B-cell numbers |
| D813 | Adenosine deaminase [ADA] deficiency |
| D816 | Major histocompatibility complex class I deficiency |
| D817 | Major histocompatibility complex class II deficiency |
| D818 | Other combined immunodeficiencies |
| D819 | Combined immunodeficiency, unspecified |
| D822 | Immunodeficiency with short-limbed stature |
| D828 | Immunodeficiency associated with other specified major defects |
| D830 | Common variable immunodeficiency |
| D831 | Common variable immunodeficiency with predominant immunoregulatory T-cell disorders |
| D832 | Common variable immunodeficiency with autoantibodies to B- or T-cells |
| D838 | Other common variable immunodeficiencies |
| D839 | Common variable immunodeficiency, unspecified |
| D848 | Other specified immunodeficiencies |
| D849 | Immunodeficiency, unspecified |
| Z94802 | Allogeneic hematopoietic stem-cell transplantation, bone marrow transplant, related donor |
| Z94804 | Allogeneic hematopoietic stem-cell transplantation, bone marrow transplant, unrelated donor |
| Z940 | Kidney transplant status |
| Z941 | Heart transplant status |
| Z942 | Lung transplant status |
| Z943 | Heart and lungs transplant status |
| Z944 | Liver transplant status |
| Z945 | Skin transplant status |
| Z946 | Bone transplant status |
| Z947 | Corneal transplant status |
| Z948 | Other transplanted organ and tissue status |
| Z94802 | Allogeneic hematopoietic stem-cell transplantation, bone marrow transplant, related donor |
| Z94804 | Allogeneic hematopoietic stem-cell transplantation, bone marrow transplant, unrelated donor |
| Z949 | Transplanted organ and tissue status, unspecified |
| B24 | Unspecified human immunodeficiency virus [HIV] disease |
| B240 | Unspecified pre-acquired immunodeficiency syndrome [AIDS] |
| B241 | Unspecified full-blown AIDS |
| B249 | Unspecified HIV infection |

1. **Hepatic diseases**

| B180 | Chronic viral hepatitis B with delta-agent |
| --- | --- |
| B181 | Chronic viral hepatitis B without delta-agent |
| B182 | Chronic viral hepatitis C |
| B189 | Chronic viral hepatitis, unspecified |
| B199 | Unspecified viral hepatitis without hepatic coma |
| C787 | Secondary malignant neoplasm of liver and intrahepatic bile duct |
| D376 | Liver, gallbladder, and bile ducts neoplasm |
| K701 | Alcoholic hepatitis |
| K703 | Alcoholic cirrhosis of liver |
| K704 | Alcoholic hepatic failure |
| K709 | Alcoholic liver disease, unspecified |
| K711 | Toxic liver disease with hepatic necrosis |
| K713 | Toxic liver disease with chronic persistent hepatitis |
| K714 | Toxic liver disease with chronic lobular hepatitis |
| K715 | Toxic liver disease with chronic active hepatitis |
| K716 | Toxic liver disease with hepatitis not elsewhere classified |
| K718 | Toxic liver disease with other disorders of liver |
| K719 | Toxic liver disease, unspecified |
| K738 | Other chronic hepatitis, not elsewhere classified |
| K739 | Chronic hepatitis, unspecified |
| K740 | Hepatic fibrosis |
| K746 | Other and unspecified cirrhosis of liver |
| K754 | Autoimmune hepatitis |
| K758 | Other specified inflammatory liver diseases |
| K759 | Inflammatory liver disease, unspecified |
| K768 | Other specified diseases of liver |
| K769 | Liver disease, unspecified |
| K831 | Obstruction of bile duct |
| P591 | Inspissated bile syndrome |
| P788 | Other specified perinatal digestive system disorder |
| Z871 | Personal history of diseases of the digestive system |

1. Obesity

| E6600 | Obesity due to excess calories in adults with a body mass index (BMI) ≥30 kg / m2 or more and < 40 kg / m2, or obesity due to excess calories in children |
| --- | --- |
| E6601 | Obesity due to excess calories in adults with BMI ≥40 kg / m2 and <50 kg / m2 |
| E6602 | Obesity due to excess calories in adults with BMI ≥50 kg / m2 |
| E6609 | Obesity due to excess calories in adults, BMI not specified |
| E6610 | Drug-induced obesity with BMI ≥30 kg / m2 and < 40 kg / m2, or drug-related childhood obesity |
| E6611 | Drug-induced obesity with BMI ≥40 kg / m2 and < 50 kg / m2 |
| E6620 | Morbid obesity with alveolar hypoventilation, with BMI ≥30 kg / m2 and < 40 kg / m2 |
| E6690 | Unspecified obesity, adult with BMI ≥30 kg / m2 and <40 kg / m2, or unclear childhood obesity |
| E6691 | Unspecified obesity, adult with BMI ≥40 kg / m2 and < 50 kg / m2 |
| E6692 | Unspecified adult obesity, with BMI ≥ 50 kg / m2 |
| E6699 | Unspecified adult obesity, with BMI not specified |

**Table S6 – Long term disease (ALD) codes for pre-existing condition at risk for severe influenza**

| ALD 1 | Stroke |
| --- | --- |
| ALD 2 | Bone marrow failures and others chronic cytopenias, myelo-dysplastic syndromes |
| ALD 5 | Chronic cardiac arrhythmia |
| ALD 6 | Chronic hepatitis and cirrhosis of liver |
| ALD 7 | Severe immunodeficiency, Human Immunodeficiency Virus infection, severe immunodeficiency |
| ALD 8 | Type 1 and 2 diabetes |
| ALD 9 | Severe neuromuscular disorders |
| ALD 10 | Sickle cell disease |
| ALD 13 | Ischemic heart disease |
| ALD 14 | Severe respiratory insufficiency due to chronic obstructive pulmonary disease |
| ALD 18 | Cystic fibrosis |
| ALD 19 | Chronic kidney disease |
| ALD 21 | Polyarteritis nodosa, lupus erythematosus, systemic sclerosis |
| ALD 22 | Rheumatoid arthritis |
| ALD 24 | Crohn disease |
| ALD 25 | Multiple sclerosis |
| ALD 27 | Ankylosing spondylitis |
| ALD 28 | Transplanted organ or tissue |
| ALD 30 | Malignant tumor, malignant neoplasm of lymphoid, hematopoietic and related tissue |

ALD: affection longue durée

**Table S7 - Anatomical therapeutic chemical drugs related to condition at risk for severe influenza**

| A 10 | Drugs used in diabetes |
| --- | --- |
| C01 | Cardiac therapy |
| C02 | Antihypertensives |
| C03 | Diuretics |
| C07 | Beta blocking agents |
| C08 | Calcium channel blockers |
| C09 | Agents acting on the renin–angiotensin system |
| L02B | Hormone antagonists and related agents |
| L04 | Immunosuppressants |
| R03 | Drugs for obstructive airway diseases |
